# The long-term cost-effectiveness of once-weekly semaglutide versus sitagliptin for the treatment of type 2 diabetes in China

**DOI:** 10.1186/s13561-024-00499-2

**Published:** 2024-04-02

**Authors:** Shuyan Gu, Jinghong Gu, Xiaoyong Wang, Xiaoling Wang, Lu Li, Hai Gu, Biao Xu

**Affiliations:** 1 Center for Health Policy and Management Studies, School of Government, Nanjing University, 163 Xianlin Road, Nanjing, 210023 Jiangsu China; 2 Department of Economics, University of Washington, Seattle, WA USA; 3 Health Insurance Office, Shandong Provincial Hospital Affiliated to Shandong First Medical University, Jinan, Shandong China; 4 Department of Endocrinology, Xinhua Hospital, Shanghai Jiao Tong University School of Medicine, Shanghai, China; 5 Department of Pharmacy, First Affiliated Hospital of Dalian Medical University, Dalian, Liaoning China

**Keywords:** Cost-effectiveness, Semaglutide, Sitagliptin, Type 2 diabetes, China

## Abstract

**Background:**

To estimate the long-term cost-effectiveness of once-weekly semaglutide versus sitagliptin as an add-on therapy for type 2 diabetes patients inadequately controlled on metformin in China, to better inform healthcare decision making.

**Methods:**

The Cardiff diabetes model which is a Monte Carlo micro-simulation model was used to project short-term effects of once-weekly semaglutide versus sitagliptin into long-term outcomes. Short-term data of patient profiles and treatment effects were derived from the 30-week SUSTAIN China trial, in which 868 type 2 diabetes patients with a mean age of 53.1 years inadequately controlled on metformin were randomized to receive once-weekly semaglutide 0.5 mg, once-weekly semaglutide 1 mg, or sitagliptin 100 mg. Costs and quality-adjusted life years (QALYs) were estimated from a healthcare system perspective at a discount rate of 5%. Univariate sensitivity analysis, scenario analysis, and probabilistic sensitivity analysis were conducted to test the uncertainty.

**Results:**

Over patients’ lifetime projections, patients in both once-weekly semaglutide 0.5 mg and 1 mg arms predicted less incidences of most vascular complications, mortality, and hypoglycemia, and lower total costs compared with those in sitagliptin arm. For an individual patient, compared with sitagliptin, once-weekly semaglutide 0.5 mg conferred a small QALY improvement of 0.08 and a lower cost of $5173, while once-weekly semaglutide 1 mg generated an incremental QALY benefit of 0.12 and a lower cost of $7142, as an add-on to metformin. Therefore, both doses of once-weekly semaglutide were considered dominant versus sitagliptin with more QALY benefits at lower costs.

**Conclusion:**

Once-weekly semaglutide may represent a cost-effective add-on therapy alternative to sitagliptin for type 2 diabetes patients inadequately controlled on metformin in China.

**Supplementary Information:**

The online version contains supplementary material available at 10.1186/s13561-024-00499-2.

## Background

There is a global epidemic of diabetes. China has the world’s largest number of diabetes population, with approximately 140.9 million people with a diagnosis, leading to associated deaths of 1.4 million and health expenditure of $165.3 billion in 2021 [1]. Type 2 diabetes as the major type of diabetes (over 90%) imposes huge disease burden on patients and healthcare systems [1]. Because healthcare resources are limited and healthcare budgets are faced with growing strain, the common goal of healthcare systems worldwide is to maximize the health across populations with limited healthcare resources [2]. Thus, choosing therapies that are both effective and cost-saving are paramount for optimizing treatment for type 2 diabetes, so as to minimize the disease burden of patients and optimize the allocation and utilization of healthcare resources. Besides, a novel therapy is sometimes not attractive in the short-term because of its high drug acquisition cost, while it may turn into cost-effective when compositely considering the long-term health events, mortality, and quality of life. Therefore, economic evaluations of novel therapies versus all competing therapies and over all relevant time frames (e.g., short-term and long-term) have become increasingly important.

Treatment targets of modern therapies for type 2 diabetes should consider composite outcomes incorporating clinical effects such as efficacy, impacts on body weight, effects on vascular complications, hypoglycemia risk, and adverse effect risks, alongside patient preferences, access and costs [3]. Incretin therapies, such as glucagon-like peptide-1 receptor agonists (GLP-1 RAs) and dipeptidyl peptidase-4 inhibitors (DPP-4is), are efficacious modern therapies for type 2 diabetes which will offer multifactorial clinical benefits. They are recommended by international and Chinese clinical guidelines as second-line add-on therapies to patients inadequately controlled on metformin monotherapy [3, 4]. Once-weekly subcutaneous semaglutide as a novel long-acting GLP-1 RA, is reported to confer reductions in blood glucose, blood pressure, and body weight, with a decreased risk of cardiovascular complications. Once-daily oral sitagliptin as the first registered representative of DPP-4is, is reported to show reductions in blood glucose and blood pressure, with a neutral effect on body weight and cardiovascular outcomes. The short-term efficacy and safety of once-weekly semaglutide and sitagliptin as an add-on therapy for a global population with type 2 diabetes were compared in the SUSTAIN 2 trial (i.e., a 56-week, randomized, double-blind, double-dummy, multicenter, multinational, parallel-group trial) and the SUSTAIN China trial (i.e., a 30-week, randomized, double-blind, double-dummy, multicenter, multiregional, parallel-group trial) [5, 6]. Once-weekly semaglutide was reported to be superior to sitagliptin in reducing blood glucose and body weight for type 2 diabetes patients inadequately controlled on metformin, thiazolidinediones, or both in the short run.

Regarding the increased health and economic tolls of type 2 diabetes and the stress of healthcare systems, it is necessary to compare the long-term clinical benefits and cost implications of once-weekly semaglutide with sitagliptin. However, there is a lack of pharmacoeconomic evidence on the two therapies in the Chinese population. Therefore, from the healthcare system perspective, this study aimed to conduct a modelling study based on the clinical data from the SUSTAIN China trial [6, 7], to project and compare the long-term cost-effectiveness of once-weekly semaglutide with sitagliptin as an add-on therapy for type 2 diabetes patients inadequate controlled on metformin in China.

## Methods

### Model overview and model setting

A published and validated mathematical model, the Cardiff diabetes model, is used. It is a patient-level, 6-monthly time increments, Monte Carlo micro-simulation model for evaluating the cost-effectiveness of comparable therapy arms in type 2 diabetes, with each arm comprised of three therapy lines. The model is not publicly available but the detailed model structure and algorithms have been published [8–14]. The model simulates the disease progression and projects the incidences of diabetes-related vascular complications and mortality based on the United Kingdom Prospective Diabetes Studies (UKPDS) 68 or 82 risk equations, combined with patient profiles and treatment effects of the therapies [13, 14]. The vascular complications include ischemic heart disease, myocardial infarction, congestive heart failure, stroke, amputation, blindness, end-stage renal disease, and ulcer. The patient profiles include patient characteristics (e.g., age, diabetes duration) and clinical risk factors (e.g., hemoglobin Alc [HbA1c], systolic blood pressure [SBP], and body weight). The natural progressions of HbA1c and SBP are modeled via the implementation of UKPDS 68 risk equations, and that of body weight is modeled linearly based on a weight gain of 0.1 kg per year by default.

The patient profiles of the initial cohort, treatment effects and pharmacy costs of the therapies, and medical costs and utility changes associated with diabetes-related events were key input parameters required by the model. In the base-case analyses, the UKPDS 82 risk equations with a 40-year time horizon were used to simulate and capture the whole disease progression of vascular complications; and a discount rate of 5% was used for projected costs and benefits in line with China guidelines [15]. Outcomes including the quality-adjusted life years (QALYs), total costs, and incremental cost-effectiveness ratios (ICERs) were key outputs of the model.

### Patient cohort and patient profiles

An initial cohort of 10,000 Chinese adult with type 2 diabetes inadequately controlled on metformin monotherapy was established. Baseline patient profiles of the cohort were mainly sourced from the full population in the SUSTAIN China trial [6, 7], which targeted at 868 type 2 diabetes patients (≥ 18 years) inadequately controlled (HbA1c 7.0-10.5%) despite treated with metformin monotherapy at a stable dose of 1500 mg or a maximum tolerated dose of 1000 mg thus require treatment intensification. The trial enrolled about 70% (605/868) of the patients in China, with the remaining patients from Brazil, Korea, South Africa, and Ukraine. Data unavailable in the SUSTAIN China trial such as smokers’ proportion and cholesterol level were assumed to be the same as the general Chinese type 2 diabetes population and supplemented from a nationwide cohort study of type 2 diabetes in China [16] (Table 1).

**Table 1:** Model inputs: patient profiles, treatment effects, costs, and utility changes

**Patient Profiles**	**The initial cohort**				**Source**						
**Patient characteristics**	Mean		SE								
Age, year	53.10		0.37		[6, 7]						
Female proportion, %	43		2								
Diabetes duration, year	6.38		0.18								
Height, m ^†^	1.66		–								
Afro-Caribbean proportion, %	3		1								
Indian proportion, %	0		0								
Smokers proportion, %	22		1		[16]						
**Clinical risk factors**	**Mean**		**SE**								
HbA1c, %	8.10		0.03		[6]						
TC, mg/dl ^‡^	193.05		0.66		[16]						
HDL-C, mg/dl ^‡^	46.33		0.20								
LDL-C, mg/dl ^‡^	111.97		0.51								
SBP, mmHg	128.80		0.50		[6]						
Body weight, kg	76.40		0.54								
**Effect and costs with treatments**	**Semaglutide 0.5 mg + metformin**		**Semaglutide 1 mg + metformin**		**Sitagliptin 100 mg + metformin**		**Source**	**Insulin**	**Source**		
**Treatment effects**	**Mean**	**SE**	**Mean**	**SE**	**Mean**	**SE**		**Mean**			
HbA1c change, %	–1.40	0.06	–1.70	0.06	–0.90	0.05	[6, 7]	−1.11	[18]		
SBP change, mmHg	–3.40	0.87	–6.60	0.79	–1.10	0.80		0			
Weight change, kg	–2.90	0.22	–4.20	0.22	–0.40	0.15		1.9			
Symptomatic hypoglycemia, %	0.70	0.49	2.07	0.84	1.38	0.68		62			
Severe hypoglycemia, %	0	0	0	0	0	0		2			
Gastrointestinal reactions, %	37.63	2.86	44.48	2.92	18.97	2.30		0			
Discontinuation, %	12.54	1.96	15.86	2.15	5.86	1.38		0			
**Treatment costs**	**Mean**		**Mean**		**Mean**						
Annual costs, ¥ ($) ^§^	6042 (897)		9711 (1441)		2695 (400)		[20–22]				
**Costs and utility with diabetes related events**	**Fatal costs**, **¥ ($)** ^§^		**Non-fatal costs**, **¥ ($)** ^§^		**Maintenance costs, ¥ ($)** ^§^		**Source**	**Utility change**			**Source**
	**Mean**	**SE**	**Mean**	**SE**	**Mean**	**SE**		**Mean**	**SE**		
Ischemic heart disease	22,775 (3381)	6714 (997)	25,188 (3739)	1664 (247)	3476 (516)	459 (68)	[12]	–0.028	0.005		[24]
Myocardial infarction	41,660 (6184)	8826 (1310)	56,088 (8325)	3985 (591)	6907 (1025)	–		–0.028	0.005		
Congestive heart failure	37,498 (5566)	13,930 (2068)	34,277 (5088)	6667 (990)	3286 (488)	1898 (282)		–0.028	0.005		
Stroke	73,290 (10,879)	19,838 (2945)	26,883 (3990)	2553 (379)	4512 (670)	776 (115)		–0.101	0.006		
Amputation	–	–	23,522 (3491)	–	3739 (555)	–		–0.118	0.009		
Blindness	–	–	16,728 (2483)	652 (97)	5518 (819)	512 (76)		–0.022	0.005		
End-stage renal disease	16,396 (2434)	3922 (582)	16,893 (2508)	585 (87)	5907 (877)	574 (85)		–0.058	0.006		
Ulcer	–	–	26,542 (3940)	2868 (426)	6188 (919)	1629 (242)		–0.118	0.009		
Symptomatic hypoglycemia	–	–	0	–	–	–		–0.007	0.002		
Severe hypoglycemia	–	–	4345 (645)	–	–	–	[23]	–0.008	0.004		
Gastrointestinal reactions	–	–	0	–	–	–		–0.034	0		[25]
BMI per unit increase	–	–	–	–	–	–		–0.0061	0.001		[26]
BMI per unit decrease	–	–	–	–	–	–		0.0061	0.001		
**BMI-related costs**	**Costs, ¥ ($)** ^§^		**BMI**	**Costs, ¥ ($)** ^§^		**BMI**	**Costs, ¥ ($)** ^§^			**Source**	
≤ 23	0		29	15,022 (2230)		35	30,881 (4584)			[12]	
24	1807 (268)		30	17,665 (2622)		36	33,524 (4976)				
25	4450 (660)		31	20,308 (3014)		37	36,167 (5368)				
26	7093 (1053)		32	22,951 (3407)		38	38,810 (5761)				
27	9736 (1445)		33	25,595 (3799)		39	41,453 (6153)				
28	12,379 (1837)		34	28,238 (4191)		40+	44,096 (6545)				

BMI body mass index, HbA1c hemoglobin Alc, HDL-C high-density lipoprotein cholesterol, LDL-C low-density lipoprotein-cholesterol, SBP systolic blood pressure, SE standard error, TC total cholesterol

^†^ As only BMI and body weight of the patients were reported, height was calculated by: sqrt (weight/BMI).

^‡^ The unit of cholesterol was converted by: 1 mg/dl = 0.0259 mmol/l [47]

^§^ For the costs, data are 2022 Chinese yuan, ¥ (2022 US dollar, $), and are presented in the form of rounding to nearest whole number. One US dollar was equal to ¥6.737 in 2022 [19]

### Treatment algorithm and treatment effects

Patients started the model simulations by receiving either once-weekly semaglutide 0.5 mg, once-weekly semaglutide 1 mg, or sitagliptin 100 mg as an add-on therapy to metformin, which was regarded as “first therapy line” in this study. In case of inadequate glucose control, therapy escalation commenced. An HbA1c level of 7.0% was used as the escalation threshold in line with the Chinese clinical guideline [4], to switch from “first therapy line” to “second therapy line” and from “second therapy line” to “third therapy line”. The insulin rescue therapy was used as both second and third therapy lines [17]. The patient ended the simulation when time-horizon was reached or death occurred. The treatment effects, adverse effects, and rates of therapy discontinuation of once-weekly semaglutide and sitagliptin as an add-on to metformin were sourced from the SUSTAIN China trial [6, 7]. The 30-week changes from baseline of biochemical outcomes including HbA1c, SBP, and body weight were used to inform clinical efficacy. Rates of hypoglycemia and gastrointestinal reactions were used to inform adverse effects [6, 7]. The treatment effects of insulin rescue therapy used the inherent insulin therapy profile of Cardiff diabetes model [18] (Table 1).

### Costs

Direct medical costs for treating type 2 diabetes and its associated events were estimated. All costs were expressed in both 2022 Chinese yuan (¥) and US dollar ($). One US dollar was equal to ¥6.737 in 2022 [19]. Annual pharmacy cost of a drug was calculated as its retail price times its annual dose. The retail prices were sourced from DRUGDATAEXPY or Sunshine Medical Procurement All-In-One (SMPA) in China [20–22]. DRUGDATAEXPY is a medicine intelligence and health industry data service provider. SMPA is an institution undertaking the daily work and implementation of national centralized drug procurement and use program. The drug doses were obtained from the SUSTAIN China trial [6]. Since a drug may have different brands and specifications and thus has different prices, the average costs of the drugs were calculated (Additional file 1 Table S1). Insulin cost per kilogram weight per day was assumed to be ¥0.137 ($0.02) based on the inherent profile of the model. Costs for treating vascular complications, cost for treating severe hypoglycemia, and body mass index (BMI)-related costs in China were sourced and estimated based on previous published studies [12, 23]. BMI-related costs relate to increased prescribing costs per BMI unit. For example, a patient with a BMI of 24 kg/m^2^ will incur a BMI associated cost of ¥1807 ($268) per year in this study. Costs for symptomatic hypoglycemia and gastrointestinal reactions were assumed to be 0, as they are usually not treated with medication and relevant published evidence was not available (Table 1).

### Utilities

Utility decrements associated with vascular complications and hypoglycemia were sourced and assumed based on a cross-sectional study in China. That study enrolled 7081 type 2 diabetes patients with and without vascular complications or comorbidities from nine cities in China, and investigated the utility decrements associated with vascular complications and comorbidities [24]. Utility changes associated with gastrointestinal reactions and BMI-related changes were abstracted from other published sources [25, 26] (Table 1).

### Sensitivity analysis

A series of sensitivity analyses including univariate sensitivity analyses, scenario analyses, and probabilistic sensitivity analyses (PSA) were carried out to assess the impact of uncertainty around model inputs. In the univariate sensitivity analyses, variations in the time horizons, discount rates, risk equations, HbA1c threshold for therapy escalation, BMI-related utility changes and costs, and utility decrements and costs associated with vascular complications were tested. Additionally, scenario analyses using the patient profiles and treatment effects of the Chinese subpopulation in the SUSTAIN China trial were conducted [6]. Scatter plots of the ICERs and cost-effectiveness acceptability curves were generated in the PSA. All sensitivity analyses were done for 10,000 patients over 40 years.

## Results

### Base-case results

All three treatment arms showed positive effects in lowering HbA1c, SBP, and body weight for type 2 diabetes patients (Additional file 1 Fig. S1). After 40-year simulation, the predicted incidences of vascular complications (except nephropathy), mortality, and hypoglycemia were lower for patients in once-weekly semaglutide 0.5 mg arm and 1 mg arm compared with those in sitagliptin arm. Correspondingly, the costs for treating most diabetes-related events were lower in once-weekly semaglutide 0.5 mg arm and 1 mg arm. Therefore, although once-weekly semaglutide 0.5 mg arm and 1 mg arm conferred higher pharmacy costs, overall, they were associated with lower total costs than sitagliptin arm (Additional file 1 Table S2).

For an individual patient, the accumulated QALYs gained over 40 years were 12.23, 12.27, and 12.15, with the total costs spent of ¥248,546 ($36,893), ¥235,279 ($34,923), and ¥283,394 ($42,065) in once-weekly semaglutide 0.5 mg arm, 1 mg arm, and sitagliptin arm, respectively. Generally, compared with sitagliptin arm, once-weekly semaglutide 0.5 mg arm conferred a small QALY improvement of 0.08 and a lower cost of ¥34,848 ($5173); while once-weekly semaglutide 1 mg arm generated an incremental QALY benefit of 0.12 and a lower cost of ¥48,115 ($7142). These results showed that both doses of once-weekly semaglutide were dominant versus sitagliptin with more QALY benefits and lower costs (Table 2).

**Table 2:** Base-case results: cost-effectiveness of once-weekly semaglutide 0.5 mg and 1 mg versus sitagliptin 100 mg when added to metformin (per patient)

	Semaglutide 0.5 mg + metformin	Semaglutide 1 mg + metformin	Sitagliptin 100 mg + metformin
Discounted QALYs	12.23	12.27	12.15
Discounted Costs, ¥ ($) ^†^	248,546 (36,893)	235,279 (34,923)	283,394 (42,065)
Incremental QALYs	0.08	0.12	–
Incremental Costs, ¥ ($) ^†^	–34,848 (–5173)	–48,115 (–7142)	–
ICER (cost per QALY gained)	Semaglutide 0.5 mg + metformin dominant ^‡^	Semaglutide 1 mg + metformin dominant ^§^	–

ICER incremental cost-effectiveness ratio, QALY quality-adjusted life year

^†^ For the costs, data are 2022 Chinese yuan, ¥ (2022 US dollar, $). One US dollar was equal to ¥6.737 in 2022 [19]

^‡^ Once-weekly semaglutide 0.5 mg + metformin dominated sitagliptin + metformin with more QALYs and lower costs

^§^ Once-weekly semaglutide 1 mg + metformin dominated sitagliptin + metformin with more QALYs and lower costs

### Sensitivity analysis results

Across the sensitivity and scenario analyses carried out around model inputs, both once-weekly semaglutide 0.5 mg and 1 mg remained dominant over sitagliptin with more QALYs and lower costs in most scenarios, except one extreme scenario considering BMI-related costs to be 0, thus the base-case findings were likely to be robust (Table 3).

**Table 3:** Sensitivity analysis results: cost-effectiveness of once-weekly semaglutide 0.5 mg and 1 mg versus sitagliptin 100 mg when added to metformin (per patient)

Scenarios	Semaglutide 0.5 mg + metformin vs. Sitagliptin 100 mg + metformin			Semaglutide 1 mg + metformin vs. Sitagliptin 100 mg + metformin		
	Incremental QALYs	Incremental Costs, ¥ ($) ^†^	ICER	Incremental QALYs	Incremental Costs, ¥ ($) ^†^	ICER
**Univariate sensitivity analyses**						
Time horizon set to be 10 years	0.04	–18,169 (–2697)	dominant ^‡^	0.05	–24,182 (–3589)	dominant ^§^
Time horizon set to be 20 years	0.06	–29,521 (–4382)	dominant ^‡^	0.09	–41,089 (–6099)	dominant ^§^
Discount rate (costs and benefits) set to be 0% in line with China guidelines	0.17	–64,940 (–9639)	dominant ^‡^	0.25	–92,752 (–13,768)	dominant ^§^
Discount rate (costs and benefits) set to be 8% in line with China guidelines	0.06	–26,049 (–3867)	dominant ^‡^	0.09	–35,081 (–5207)	dominant ^§^
Use UKPDS 68 risk equations to run model	0.08	–33,686 (–5000)	dominant ^‡^	0.12	–46,344 (–6879)	dominant ^§^
HbA1c threshold for therapy escalation set to be 7.5%	0.08	–31,392 (–4660)	dominant ^‡^	0.11	–35,916 (–5331)	dominant ^§^
Utility impacts set to be 0.017 per unit BMI decrease and − 0.047 per unit BMI increase [29]	0.56	–34,848 (–5173)	dominant ^‡^	0.81	–48,115 (–7142)	dominant ^§^
BMI-related costs set to be 0	0.08	2805 (416)	34,163 (5071)	0.12	12,125 (1800)	99,466 (14,764)
BMI-related costs halved	0.08	–16,021 (–2378)	dominant ^‡^	0.12	–17,995 (–2671)	dominant ^§^
Alternative utility impacts for vascular complications [27, 28]	0.08	–34,848 (–5173)	dominant ^‡^	0.12	–48,115 (–7142)	dominant ^§^
Costs of vascular complications halved	0.08	–34,662 (–5145)	dominant ^‡^	0.12	–47,839 (–7101)	dominant ^§^
**Scenario analyses**	**0.11**	**–37,451** **(–5559)**	dominant ^‡^	**0.15**	**–32,841** **(–4875)**	dominant ^§^
**Probabilistic sensitivity analyses**	**0.08**	**–33,297** **(–4942)**	dominant ^‡^	**0.12**	**–44,632** **(–6625)**	dominant ^§^

BMI body mass index, HbA1c hemoglobin Alc, ICER incremental cost-effectiveness ratio, QALY quality-adjusted life year

^†^ For the costs, data are 2022 Chinese yuan, ¥ (2022 US dollar, $). One US dollar was equal to ¥6.737 in 2022 [19]

^‡^ Once-weekly semaglutide 0.5 mg + metformin dominated sitagliptin + metformin with more QALYs and lower costs

^§^ Once-weekly semaglutide 1 mg + metformin dominated sitagliptin + metformin with more QALYs and lower costs

In the univariate sensitivity analyses, either when applying a time horizon of 10 to 20 years, a discount rate of 8%, or when using an HbA1c threshold of 7.5% for therapy escalation, both the QALY improvements and cost-savings gained by once-weekly semaglutide 0.5 mg arm and 1 mg arm reduced compared with that in base case. Conversely, when using a discount rate of 0%, both the QALY improvements and cost savings gained by once-weekly semaglutide 0.5 mg arm and 1 mg arm increased compared with that in base case. Meanwhile, either when using UKPDS 68 risk equations, or when altering utility impacts [27, 28] or halving costs for vascular complications, the resulted QALY and cost outcomes remained similar to that in base case. Besides, when altering BMI-related utilities (i.e., utility impacts set to be 0.017 per unit BMI decrease and − 0.047 per unit BMI increase [29]), the QALY improvements gained increased; and when halving BMI-related costs, the cost savings gained reduced, as compared with that in base case. In all above scenarios, once-weekly semaglutide 0.5 mg and 1 mg remained dominant over sitagliptin with more QALYs and lower costs. Only when BMI-related costs were set to be 0, the results changed largely: once-weekly semaglutide 0.5 mg arm and 1 mg arm costed more than sitagliptin arm with the ICERs of ¥34,163 ($5071)/QALY and ¥99,466 ($14,764)/QALY, respectively. Both were within two times GDP per capita of China as GDP per capita is ¥85,698 ($12,720) in 2022 [30], meaning that once-weekly semaglutide 0.5 mg and 1 mg were cost-effective compared with sitagliptin according to China guidelines [15].

**Fig. 1:**
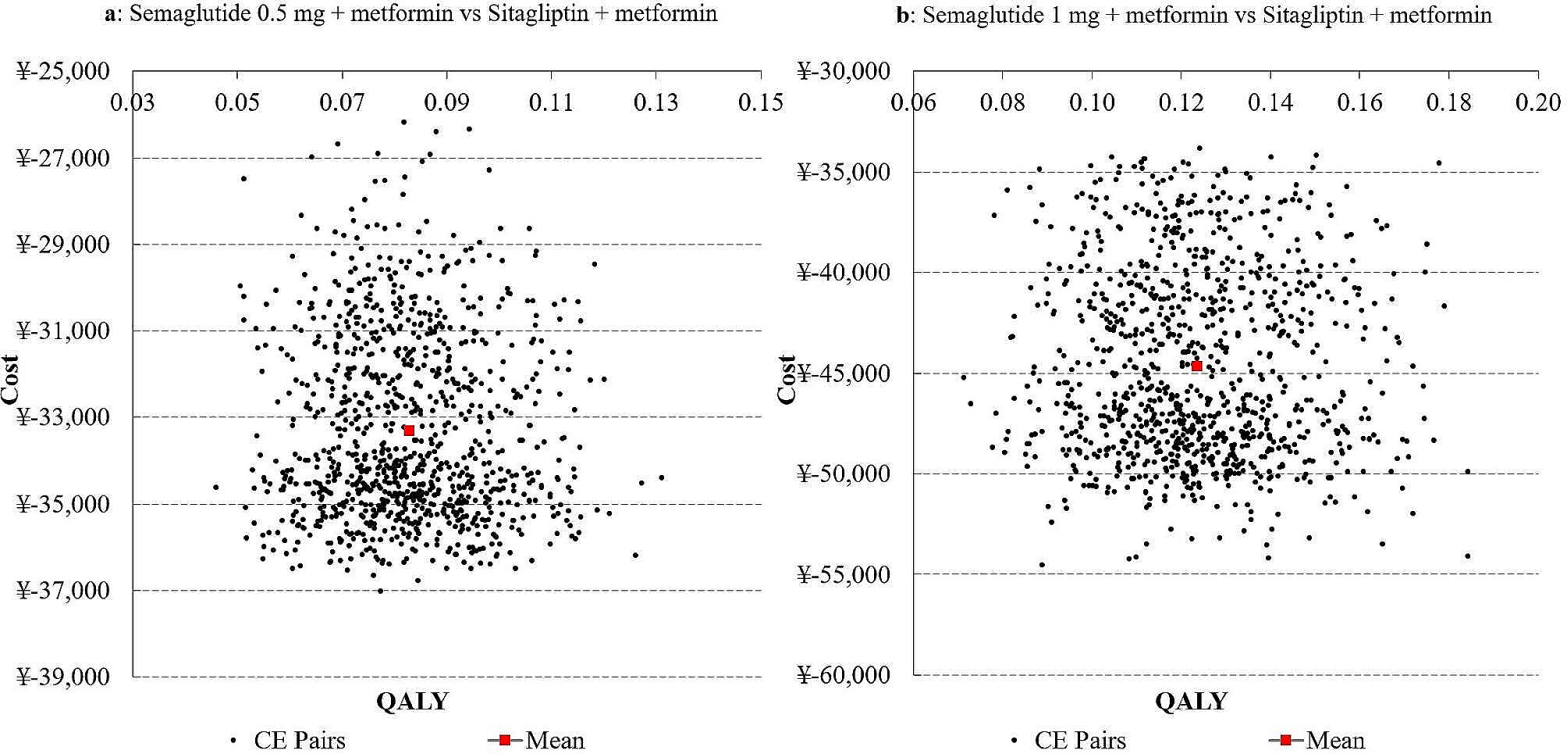
Scatter plots of the incremental cost-effectiveness ratios for once-weekly semaglutide 0.5 mg + metformin versus sitagliptin + metformin (**a**) and once-weekly semaglutide 1 mg + metformin versus sitagliptin + metformin (**b**) with a cost-effective threshold value of ¥85,698 ($12,720) (one time GDP per capita of China in 2022)

Both in the scenario analyses of using alternative patient profiles and treatment effects [6] and in the PSA, once-weekly semaglutide 0.5 mg arm and 1 mg arm remained superior to sitagliptin arm with more QALYs at lower costs. Besides, PSA showed that the probabilities of once-weekly semaglutide 0.5 mg and 1 mg to be cost-effective compared with sitagliptin were both 100%, at a strict cost-effective threshold of one time GDP per capita of China in 2022 [30] (Fig. 1). The cost-effectiveness acceptability curves were shown in Additional file 1 Fig. S2.

## Discussion

Rational drug use is the key to reduce disease burden and optimize healthcare resources allocation. It requires patients to receive drugs appropriate to their clinical needs, and the drugs should be available at the lowest costs affordable to patients and healthcare systems [31]. Thus, economic evaluations are necessary for the definition of rational drug use as they can provide cost and effectiveness evidence of competing therapies. This study provided the first comparison of long-term cost-effectiveness of once-weekly semaglutide versus sitagliptin as add-on for type 2 diabetes patients treated with metformin requiring treatment intensification in China. The results indicated that both once-weekly semaglutide 0.5 mg and 1 mg were cost-effective treatments compared with sitagliptin for type 2 diabetes, with higher QALY benefits at lower costs. The results of sensitivity analyses verified the robustness of the base-case results and assured confidence in the projections.

The prevalence of cardiovascular diseases is increasing in China, affecting about 330 million people in 2018 [32]. Type 2 diabetes patients are faced with 2- to 4-fold higher risks of cardiovascular diseases than nondiabetic individuals [33]. Cardiovascular complications impose tremendous health and economic burden on type 2 diabetes patients. They remain the leading causes of deaths in type 2 diabetes patients, accounting for about 65% of the deaths [34]. Mean utility of type 2 diabetes patients hospitalized for a vascular complication was 0.562, varying from 0.395 with amputation to 0.714 with impaired vision [35]. Medical costs of type 2 diabetes patients with vascular complications were 3.46-fold higher than that of those without vascular complications [36]. Thus, preventing or delaying the occurrences of vascular complications has become one of the key treatment targets for type 2 diabetes patients. Because hyperglycemia, obesity/overweight, hypertension, and dyslipidemia are risk factors for vascular complications in type 2 diabetes [37]. The superior efficacy of once-weekly semaglutide in reducing blood glucose and body weight compared to sitagliptin [6] is likely to produce long-term benefits regarding less incidences of vascular complications. This was demonstrated by model simulations in this study, as projections showed that both doses of once-weekly semaglutide were associated with less incidences of vascular complications and mortality than sitagliptin in the long run, resulting in more QALY benefits and lower associated costs. Previous clinical trial also has confirmed that semaglutide possesses significantly favorable cardiovascular protective effects and decreases renal events for patients [38]. The increased benefit of once-weekly semaglutide on vascular complications was one of the key drivers of its decreased total costs in this study. As lower costs for treating vascular complications in once-weekly semaglutide partially offset its higher pharmacy costs compared to sitagliptin over patients’ lifetimes.

Besides, being overweight or obese is common in type 2 diabetes patients, consisting of about 85% of all cases [39]. Weight loss can independently reduce costs and increase utility for type 2 diabetes patients [40]. Thus, the additional benefits of once-weekly semaglutide were observed through reduced BMI-related costs and increased QALYs associated with weight loss in the long-term in this study. As a results, the total cumulative costs of once-weekly semaglutide were lower while the QALYs gains were higher than sitagliptin over patients’ lifetimes. Additionally, this study also found that once-weekly semaglutide 1 mg was associated with more QALYs gains and lower total costs compared with once-weekly semaglutide 0.5 mg, which may be closely related to its superior effects on delaying intensive treatment with insulin and reducing more body weight and the occurrences of vascular complications for patients.

To date, this study is the first to estimate the long-term cost-effectiveness of once-weekly semaglutide versus sitagliptin for type 2 diabetes patients in the Chinese setting. Only two published cost-effectiveness studies on the same comparison were found in the other settings. Both were targeted on the Spanish patients by using the clinical data from the SUSTAIN 2 trial, which included type 2 diabetes patients (≥ 18 years) inadequately controlled (HbA1c 7.0-10.5%) despite stable treatment with metformin, thiazolidinediones, or both, with female proportion of semaglutide population being 50%, mean age being 55.42 years, mean diabetes duration being 6.52 years, and mean HbA1c being 8.0% [5]. One study was a short-term cost-effectiveness analysis conducted over one year [41]. It found that compared with sitagliptin, annual medication costs per patient of once-weekly semaglutide 0.5 mg and 1 mg were marginally higher for achieving the HbA1c < 7.0% endpoint (incremental costs: EUR 514 and EUR 326), and comparable for the HbA1c < 7.0% without hypoglycemia and without weight gain endpoints (incremental costs: EUR 297 and EUR 31), but substantially lower for the ≥ 1.0% HbA1c reduction with ≥ 5.0% weight loss endpoints (incremental costs: EUR − 1253 and EUR − 2385). Both doses of semaglutide conferred comparable or lower costs versus sitagliptin when considering composite endpoints incorporating hypoglycemia and weight loss alongside glucose control [41]. The other study is a long-term cost-effectiveness analysis conducted over patients’ lifetimes using the IQVIA CORE Diabetes Model [42]. It showed that compared with sitagliptin, once-weekly semaglutide 0.5 mg and 1 mg were projected to reduce the incidences and delay the time to onsets of vascular complications, resulting in QALY improvements of 0.16 and 0.23 at cost savings of EUR 6 and EUR 692, respectively. Both doses of once-weekly semaglutide thereby were considered dominant (more effective and less costly) from a healthcare payer perspective [42]. As medical policies and national conditions vary across countries, and the background therapy and the patient profiles (e.g., the female proportion of semaglutide population in our study was 46%, mean age was 53 years, and mean HbA1c was 8.1%) in our study were not the same as the above two studies, cost-effectiveness results from our study and above two studies may be not directly comparable. Nevertheless, the results of the above studies have somewhat supported the findings of our study. Our study provided additional data in the Chinese setting, complementing the previous evidence on once-weekly semaglutide versus sitagliptin of foreign populations, and provided insights to assist decision makers in choosing between the two drugs based on their value for money. Moreover, our study also supplemented the previous cost-effectiveness analyses of once-weekly semaglutide versus other GLP-1 RAs in China, which reported that once-weekly semaglutide was dominant versus polyethylene glycol loxenatide, and was cost-effective or even dominant versus dulaglutide for type 2 diabetes patients uncontrolled on metformin after semaglutide was included in the national reimbursement drug list through price negotiation [43–45]. Our study contributed to current evidence on the economic value of once-weekly semaglutide in China, to help decision makers gain a comprehensive understanding of this drug.

There were several limitations in this study. First, as there is no long-term clinical follow-up data of the targeted drugs, this study used Cardiff diabetes model to run lifelong simulations based on the clinical data integrated from the 30-week SUSTAIN China trial. The projection of long-term outcomes from short-term data was a limitation. However, this is an inherent tenet in all long-term pharmacoeconomic modelling studies, and is the best choice to better inform healthcare decision making in the absence of long-term data recommended by China guideline for economic evaluations and computer modelling guidance for diabetes [15, 46]. Second, since no published risk equations based on the Chinese population with type 2 diabetes were found, this study used UKPDS risk equations to simulate long-term outcomes of targeted drugs. The equations are very often used in health economic models in type 2 diabetes, but as they were based on a UK population, the results might not accurately mirror the outcomes in real-world settings in China. Third, no China-specific utility decrements for blindness, end-stage renal disease, gastrointestinal reactions, and BMI changes were available. Thus, we conservatively used that of retinopathy and nephropathy in Chinese patients as alternatives, which may underestimate the impact of both complications. Besides, we used utility data of gastrointestinal reactions and BMI changes from foreign populations, which may introduce a potential bias. Lastly, total costs may be underestimated due to the unavailability of costs associated with gastrointestinal reactions, which may somewhat undermine the comparability of the treatment arms.

## Conclusion

Based on long-term projections, once-weekly semaglutide may be a cost-effective option alternative to sitagliptin as an add-on therapy to type 2 diabetes patients inadequately controlled on metformin in China, with improved clinical benefits at decreased costs. This may address an excess of medical needs in type 2 diabetes patients with a high risk of cardiovascular events, or seeking to minimize weight gain or reduce weight in treatment meanwhile reduce the associated disease burden. Our results can be used to improve clinical guidelines for the use of once-weekly semaglutide, promote the rational allocation of healthcare resources, and provide evidence for assisting in the prioritization of government reimbursements and health insurance.

### Electronic supplementary material

Below is the link to the electronic supplementary material.

Supplementary Material 1

Supplementary Material 2

## Data Availability

The datasets used and/or analyzed during this study are available from the first and corresponding authors on reasonable request.

## Abbreviations

BMI: Body mass index
DPP-4is: Dipeptidyl peptidase-4 inhibitors
GLP-1 RAs: Glucagon-like peptide-1 receptor agonists
HbA1c: Hemoglobin Alc
ICERs: Incremental cost-effectiveness ratios
PSA: Probabilistic sensitivity analyses
QALYs: Quality-adjusted life years
SBP: Systolic blood pressure
SMPA: Sunshine Medical Procurement All-In-One
UKPDS: United Kingdom Prospective Diabetes Studies

## References

[1] International Diabetes Federation. IDF Diabetes Atlas 2021 (10th edition). Available at: https://diabetesatlas.org/atlas/tenth-edition/. Accessed 28 Nov 2022. 35914061

[2] Hoomans T, Severens JL (2014). Economic evaluation of implementation strategies in health care. Implement Sci.

[3] American Diabetes Association Professional Practice C (2022). 9. Pharmacologic approaches to Glycemic Treatment: standards of Medical Care in Diabetes-2022. Diabetes Care.

[4] Chinese Diabetes Society (2021). Guideline for the prevention and treatment of type 2 diabetes mellitus in China (2020 edition). Chin J Diabetes Mellitus.

[5] Ahren B, Masmiquel L, Kumar H (2017). Efficacy and safety of once-weekly semaglutide versus once-daily sitagliptin as an add-on to metformin, thiazolidinediones, or both, in patients with type 2 diabetes (SUSTAIN 2): a 56-week, double-blind, phase 3a, randomised trial. Lancet Diabetes Endo.

[6] Ji LN, Dong XL, Li YM (2021). Efficacy and safety of once-weekly semaglutide versus once-daily sitagliptin as add-on to metformin in patients with type 2 diabetes in SUSTAIN China: a 30-week, double-blind, phase 3a, randomized trial. Diabetes Obes Metab.

[7] ClinicalTrials. Efficacy and safety of semaglutide once-weekly versus sitagliptin once-daily as add-on to metformin in subjects with type 2 diabetes (SUSTAIN - CHINA MRCT) (SUSTAIN). Available at: https://clinicaltrials.gov/ct2/show/results/NCT03061214. Accessed 27 Nov 2022.

[8] McEwan P, Peters JR, Bergenheim K, Currie CJ (2006). Evaluation of the costs and outcomes from changes in risk factors in type 2 diabetes using the Cardiff stochastic simulation cost-utility model (DiabForecaster). Curr Med Res Opin.

[9] McEwan P, Evans M, Bergenheim K (2010). A population model evaluating the costs and benefits associated with different oral treatment strategies in people with type 2 diabetes. Diabetes Obes Metab.

[10] McEwan P, Evans M, Kan H, Bergenheim K (2010). Understanding the inter-relationship between improved glycaemic control, hypoglycaemia and weight change within a long-term economic model. Diabetes Obes Metab.

[11] McEwan P, Bergenheim K, Yuan Y, Tetlow AP, Gordon JP (2010). Assessing the relationship between computational speed and Precision A Case Study comparing an interpreted versus compiled Programming Language using a Stochastic Simulation Model in Diabetes Care. PharmacoEconomics.

[12] Gu SY, Shi LZ, Shao H (2020). Choice across 10 pharmacologic combination strategies for type 2 diabetes: a cost-effectiveness analysis. Bmc Med.

[13] Clarke PM, Gray AM, Briggs A (2004). A model to estimate the lifetime health outcomes of patients with type 2 diabetes: the United Kingdom prospective diabetes study (UKPDS) outcomes Model (UKPDS 68). Diabetologia.

[14] Hayes AJ, Leal J, Gray AM, Holman RR, Clarke PM (2013). UKPDS outcomes Model 2: a new version of a model to simulate lifetime health outcomes of patients with type 2 diabetes mellitus using data from the 30 year United Kingdom prospective diabetes study: UKPDS 82. Diabetologia.

[15] Liu GG (2020). China guidelines for pharmacoeconomic evaluations (2020 Chinese-English version).

[16] Cai X, Hu D, Pan C (2020). Evaluation of effectiveness of treatment paradigm for newly diagnosed type 2 diabetes patients in Chin: a nationwide prospective cohort study. J Diabetes Investig.

[17] Gu S, Wang X, Qiao Q, Gao W, Wang J, Dong H (2017). Cost-effectiveness of exenatide twice daily vs insulin glargine as add-on therapy to oral antidiabetic agents in patients with type 2 diabetes in China. Diabetes Obes Metabolism.

[18] Waugh N, Cummins E, Royle P (2010). Newer agents for blood glucose control in type 2 diabetes: systematic review and economic evaluation. Health Technol Assess.

[19] The Organisation for Economic Co-operation and Development (OECD). Exchange rates. Available at: https://data.oecd.org/conversion/exchange-rates.htm. Accessed 25 Jan 2023.

[20] Official drug price for semaglutide. Available at: https://db.yaozh.com/yaopinzhongbiao?comprehensivesearchcontent=Semaglutide&. Accessed 27 Nov 2022.

[21] Official drug price for sitagliptin. Available at: https://db.yaozh.com/yaopinzhongbiao?comprehensivesearchcontent=Sitagliptin&. Accessed 27 Nov 2022.

[22] Official drug price for metformin. Available at: https://www.smpaa.cn/gjsdcg/2020/08/24/9560.shtml. Accessed 27 Nov 2022.

[23] Zheng Y, Wu J, Xie K (2012). Incidence and cost of hypoglycemia episode in patients with type 2 diabetes mellitus (T2DM). Chin Rural Health Service Adm.

[24] Zhang Y, Wu J, Chen Y, Shi L (2020). EQ-5D-3L decrements by diabetes complications and comorbidities in China. Diabetes Ther.

[25] Rajan N, Boye KS, Gibbs M (2016). Utilities for type 2 diabetes treatment-related attributes in a South Korean and Taiwanese Population. Value Health Reg Issues.

[26] Bagust A, Beale S (2005). Modelling EuroQol health-related utility values for diabetic complications from CODE-2 data. Health Econ.

[27] Clarke P, Gray A, Holman R (2002). Estimating utility values for health states of type 2 diabetic patients using the EQ-5D (UKPDS 62). Med Decis Mak.

[28] Currie CJ, McEwan P, Peters JR, Patel TC, Dixon S (2005). The routine collation of health outcomes data from hospital treated subjects in the Health Outcomes Data Repository (HODaR): descriptive analysis from the first 20,000 subjects. Value Health.

[29] Lane S, Levy AR, Mukherjee J, Sambrook J, Tildesley H (2014). The impact on utilities of differences in body weight among Canadian patients with type 2 diabetes. Curr Med Res Opin.

[30] National Bureau of Statistics of China. Gross Domestic Product (GDP) per capita. Available at: https://data.stats.gov.cn/easyquery.htm?cn=C01&zb=A0201&sj=2022. Accessed 11 Mar 2023.

[31] World Health Organization. Drug and therapeutics committees: a practical guide. Available at: https://apps.who.int/iris/handle/10665/68553. Accessed 27 Nov 2022.

[32] The Writing Committee of the Report on Cardiovascular Health and Diseases in China (2021). Report on Cardiovascular Health and diseases Burden in China: an updated Summary of 2020. Chin Circulation J.

[33] Tentolouris A, Eleftheriadou I, Athanasakis K (2020). Prevalence of diabetes mellitus as well as cardiac and other main comorbidities in a representative sample of the adult Greek population in comparison with the general population. Hell J Cardiol.

[34] Grundy SM, Benjamin IJ, Burke GL (1999). Diabetes and cardiovascular disease - A statement for healthcare professionals from the American Heart Association. Circulation.

[35] Gu S, Wang X, Shi L (2020). Health-related quality of life of type 2 diabetes patients hospitalized for a diabetes-related complication. Qual Life Res.

[36] Wu H, Eggleston KN, Zhong J (2019). Direct medical cost of diabetes in rural China using electronic insurance claims data and diabetes management data. J Diabetes Investig.

[37] American Diabetes Association Professional Practice C (2022). 10. Cardiovascular Disease and Risk Management: standards of Medical Care in Diabetes-2022. Diabetes Care.

[38] Ipp E, Genter P, Childress K (2017). Semaglutide and Cardiovascular outcomes in patients with type 2 diabetes. N Engl J Med.

[39] Eberhardt MS, Ogden C, Engelgau M, Cadwell B, Hedley AA, Saydah SH (2004). Prevalence of overweight and obesity among adults with diagnosed diabetes - United States, 1988–1994 and 1999–2002. MMWR Morb Mortal Wkly Rep.

[40] Nichols GA, Bell K, Kimes TM, O’Keeffe-Rosetti M (2016). Medical Care costs Associated with Long-Term Weight maintenance Versus Weight Gain among patients with type 2 diabetes. Diabetes Care.

[41] Vidal J, Malkin SJP, Hunt B, Martin V, Hallen N, Ortega FJ (2020). The short-term cost-effectiveness of once-weekly Semaglutide Versus once-Daily Sitagliptin and once-Weekly Dulaglutide for the treatment of patients with type 2 diabetes: a cost of control analysis in Spain. Diabetes Ther.

[42] Martin V, Vidal J, Malkin SJP, Hallen N, Hunt B (2020). Evaluation of the long-term cost-effectiveness of once-weekly Semaglutide Versus Dulaglutide and Sitagliptin in the Spanish setting. Adv Ther.

[43] Liu L, Ruan Z, Ung COL, et al. Long-term cost-effectiveness of subcutaneous once-weekly semaglutide versus polyethylene glycol loxenatide for treatment of type 2 diabetes mellitus in China. Diabetes Ther. 2023;14:93–107. 10.1007/s13300-022-01336-7 PMC9880095 36414806

[44] Hu S, Gu S, Qi C (2023). Cost-utility analysis of semaglutide for type 2 diabetes after its addition to the National Medical Insurance System in China. Diabetes Obes Metab.

[45] Ruan Z, Ung COL, Shen Y (2022). Long-term cost-effectiveness analysis of once-weekly Semaglutide versus Dulaglutide in patients with type 2 diabetes with inadequate Glycemic Control in China. Diabetes Ther.

[46] American Diabetes Association Consensus P (2004). Guidelines for computer modeling of diabetes and its complications. Diabetes Care.

[47] Joint committee on revision of standards of medical care for adult dyslipidemia in China (2016). Standards of medical care for adult dyslipidemia in China (2016 version). Chin Circulation J.

