# Supplementary material for: The long-term cost-effectiveness of once-weekly semaglutide versus sitagliptin for the treatment of type 2 diabetes in China
Source: Health Econ Rev. 2024 Apr 2;14:26. doi: 10.1186/s13561-024-00499-2 (PMC10988849; doi:10.1186/s13561-024-00499-2)
Supplement: Supplementary file 2 — Supplementary Material 2 [file 13561_2024_499_MOESM2_ESM.pdf]

The long-term cost-effectiveness of once-weekly semaglutide versus sitagliptin for the treatment of type 2 diabetes in China

Shuyan Gu[1], Jinghong Gu[2], Xiaoyong Wang[3], Xiaoling Wang[4], Lu Li[5], Hai Gu[1*], Biao Xu[1*]

[1] Center for Health Policy and Management Studies, School of Government, Nanjing University, Nanjing, Jiangsu, China

[2] Department of Economics, University of Washington, Seattle, WA, USA

[3] Health Insurance Office, Shandong Provincial Hospital Affiliated to Shandong First Medical University, Jinan, Shandong, China

[4] Department of Endocrinology, Xinhua Hospital, Shanghai Jiao Tong University School of Medicine, Shanghai, China

[5] Department of Pharmacy, First Affiliated Hospital of Dalian Medical University, Dalian, Liaoning, China

[*] Corresponding author: Prof Biao Xu, Nanjing University, 163 Xianlin Road, Nanjing, 210023, China (Email: xubiao@nju.edu.cn). Prof Hai Gu, Nanjing University, 163 Xianlin Road, Nanjing, 210023, China (Email: ghai1008@vip.sina.com).

Table S1 Annual pharmacy costs of the drugs

**Annual pharmacy cost of once-weekly semaglutide 0.5 mg**									
**Drug**	**Manufacturer**	**Form**	**Specification**	**Price, ¥**	**Province**	**Usage and dosage**	**Annual dose, mg**	**Annual cost, ¥**	**Mean annual cost, ¥**
Semaglutide Injection	Novo Nordisk A/S	Injection	1.34mg/ml, 1.5ml	478.8	All provinces	once weekly: 0.25 mg for 0-4 weeks followed by 0.5 mg for 5-52 weeks	25	5955.22	**5955.22**
**Annual pharmacy cost of once-weekly semaglutide 1 mg**									
**Drug**	**Manufacturer**	**Form**	**Specification**	**Price, ¥**	**Province**	**Usage and dosage**	**Annual dose, mg**	**Annual cost, ¥**	**Mean annual cost, ¥**
Semaglutide Injection	Novo Nordisk A/S	Injection	1.34mg/ml, 1.5ml	478.8	All provinces	once weekly: 0.25 mg for 0-4 weeks followed by 0.5 mg for 5-8 weeks	3	714.63	**9623.64**
Semaglutide Injection	Novo Nordisk A/S	Injection	1.34mg/ml, 3ml	813.96	All provinces	once weekly: 1.0 mg for 9-52 weeks	44	8909.01	
**Annual pharmacy cost of once-daily sitagliptin 100 mg**									
**Drug**	**Manufacturer**	**Form**	**Specification**	**Price, ¥**	**Province**	**Usage and dosage**	**Annual dose, mg**	**Annual cost, ¥**	**Mean annual cost, ¥**
Sitagliptin Phosphate Tablet	CTTQ Pharmaceutical Group Co., Ltd.	Tablet	100mg*14 tablets	95	Shanxi	once daily: 100 mg for one year	36500	2476.79	**2607.58**
Sitagliptin Phosphate Tablet	CTTQ Pharmaceutical Group Co., Ltd.	Tablet	100mg*1 tablet	6.7857	Guizhou		36500	2476.78	
Sitagliptin Phosphate Tablet	Cisen Pharmaceutical Co., Ltd.	Tablet	100mg*1 tablet	7.3758	Guizhou		36500	2692.17	
Sitagliptin Phosphate Tablet	CTTQ Pharmaceutical Group Co., Ltd.	Film-coated tablet	100mg*14 tablets	95	Guangxi		36500	2476.79	
Sitagliptin Phosphate Tablet	CTTQ Pharmaceutical Group Co., Ltd.	Tablet	100mg*14 tablets	95.0012	Ningxia		36500	2476.82	
Sitagliptin Phosphate Tablet	CTTQ Pharmaceutical Group Co., Ltd.	Tablet	100mg*14 tablets	95	Hubei		36500	2476.79	
Sitagliptin Phosphate Tablet	Merck Sharp & Dohme Ltd.	Tablet	100mg*1 tablet	7.3757	Hunan		36500	2692.13	

Sitagliptin Phosphate Tablet	Merck Sharp & Dohme Ltd.	Tablet	100mg*7 tablets	51.63	Tibet		36500	2692.14	
Sitagliptin Phosphate Tablet	Merck Sharp & Dohme Ltd.	Tablet	100mg*14 tablets	103.26	Tibet		36500	2692.14	
Sitagliptin Phosphate Tablet	Merck Sharp & Dohme Ltd.	Film-coated tablet	100mg*7 tablets	51.63	Jiangsu		36500	2692.14	
Sitagliptin Phosphate Tablet	CTTQ Pharmaceutical Group Co., Ltd.	Film-coated tablet	100mg*14 tablets	95	Jiangsu		36500	2476.79	
Sitagliptin Phosphate Tablet	CTTQ Pharmaceutical Group Co., Ltd.	Tablet	100mg*14 tablets	95	Gansu		36500	2476.79	
Sitagliptin Phosphate Tablet	Merck Sharp & Dohme Ltd.	Tablet	100mg*14 tablets	103.26	Ningxia		36500	2692.14	
Sitagliptin Phosphate Tablet	Merck Sharp & Dohme Ltd.	Film-coated tablet	100mg*7 tablets	51.63	Hubei		36500	2692.14	
Sitagliptin Phosphate Tablet	Merck Sharp & Dohme Ltd.	Tablet	100mg*14 tablets	103.28	Gansu		36500	2692.66	
Sitagliptin Phosphate Tablet	Merck Sharp & Dohme Ltd.	Tablet	100mg*7 tablets	51.63	Guizhou		36500	2692.14	
Sitagliptin Phosphate Tablet	Merck Sharp & Dohme Ltd.	Tablet	100mg*1 tablet	7.5657	Heilongjiang		36500	2761.48	
**Annual pharmacy cost of once-daily metformin 1500 mg**									
**Drug**	**Manufacturer**	**Form**	**Specification**	**Price, ¥**	**Province**	**Usage and dosage**	**Annual dose, mg**	**Annual cost, ¥**	**Mean annual cost, ¥**
Metformin Hydrochloride Sustained-release Tablet	Topfond Pharmaceutical Co., Ltd	Sustained-release tablet	0.5g*64 tablets	4.35	Liaoning, Jilin, Anhui, Fujian, Henan	once-daily: a stable dose of 1500 mg for one year	547500	74.43	**87.18**
Metformin Hydrochloride Sustained-release Tablet	Beijing Wanhui Double-Crane Pharmaceutical Co., Ltd	Sustained-release tablet	0.5g*10 tablets	0.78	Inner Mongolia, Heilongjiang, Shanghai, Hunan		547500	85.41	
Metformin Hydrochloride Sustained-release Tablet	CSPC Pharmaceutical Group Co., Ltd.	Sustained-release tablet	0.5g*60 tablets	5.13	Beijing, Shandong, Hainan, Gansu		547500	93.62	
Metformin Hydrochloride Sustained-release Tablet	Shijiazhuang Huaxin Pharmaceutical Co., Ltd.	Sustained-release tablet	0.5g*30 tablets	2.85	Tianjin, Guangxi, Sichuan, Ningxia		547500	104.03	
Metformin Hydrochloride Sustained-release Tablet	Jiangsu Deyuan Pharmaceutical Co., Ltd.	Sustained-release tablet	0.5g*30 tablets	2.97	Hebei, Shanxi, Chongqing, Qinghai		547500	108.41	
Metformin Hydrochloride Sustained-release Tablet	Nanjing Yihua Pharmaceutical Co., Ltd.	Sustained-release tablet	0.5g*36 tablets	4.1	Zhejiang, Jiangxi, Yunnan, Xizang, Tibet		547500	124.71	

Metformin Hydrochloride Sustained-release Tablet	Guangdong Sinocorp Pharmaceutical Co., Ltd.	Sustained-release tablet	0.5g*60 tablets	7.18	Hubei, Guangdong, Guizhou		547500	131.04	
Metformin Hydrochloride Sustained-release Tablet	YouCare Pharmaceutical Group Co., Ltd.	Sustained-release tablet	0.5g*60 tablets	7.68	Jiangsu, Shaanxi, Xinjiang		547500	140.16	
Metformin Hydrochloride Tablet	Chongqing Cory Pharmaceutical Co., Ltd.	Tablet	0.25g*84 tablets	1.29	Tianjin, Hainan, Chongqing, Sichuan, Guizhou		547500	33.63	
Metformin Hydrochloride Tablet	Beijing Jingfeng Pharmaceutical Group Co. Ltd.	Tablet	0.25g*100 tablets	2.94	Inner Mongolia, Shanghai, Guangdong, Xinjiang		547500	64.39	
Metformin Hydrochloride Tablet	Shanghai Sine Tianping Pharmaceutical Co.,Ltd.	Tablet	0.25g*60 tablets	1.8	Heilongjiang, Zhejiang, Henan, Shaanxi		547500	65.70	
Metformin Hydrochloride Tablet	CSPC Pharmaceutical Group Co., Ltd.	Tablet	0.5g*60 tablets	3.4	Liaoning, Fujian, Shandong, Ningxia		547500	62.05	
Metformin Hydrochloride Tablet	Penglai Nuokang Pharmaceutical Co., Ltd.	Tablet	0.25g*100 tablets	3.56	Beijing, Jilin, Hubei, Qinghai		547500	77.96	
Metformin Hydrochloride Tablet	Shijiazhuang Yiling Pharmaceutical Co., Ltd.	Tablet	0.25g*100 tablets	3.57	Shanxi, Anhui, Hunan, Tibet		547500	78.18	
Metformin Hydrochloride Tablet	Hebei Tiancheng Pharmaceutical Co., Ltd.	Tablet	0.25g*120 tablets	4.37	Hebei, Guangxi, Yunnan		547500	79.75	
Metformin Hydrochloride Tablet	Huabei Pharmaceutical Co. Ltd.	Tablet	0.5g*36 tablets	2.35	Jiangsu, Jiangxi, Gansu		547500	71.48	

Official drug price for semaglutide was obtained from https://db.yaozh.com/yaopinzhongbiao?comprehensivesearchcontent=Semaglutide&, on November 27, 2022.
Official drug price for sitagliptin was obtained from https://db.yaozh.com/yaopinzhongbiao?comprehensivesearchcontent=Sitagliptin&, on November 27, 2022.
Official drug price for metformin was obtained from https://www.smpaa.cn/gjsdcg/2020/08/24/9560.shtml, on November 27, 2022.
One year was counted as 365 days.

Table S2 Detailed base-case results: cost-effectiveness of once-weekly semaglutide 0.5 mg and 1 mg versus once-daily sitagliptin when added to metformin

**Total Events Predicted**	**Arm 1: Sitagliptin 100 mg +metformin**	**Arm 2: Semaglutide 0.5 mg +metformin**	**Arm 3: Semaglutide 1 mg +metformin**	**Difference: Arm 2 vs. Arm 1**	**Difference: Arm 3 vs. Arm 1**
**Macrovascular**					
Ischemic heart disease	2103.36	2100.96	2098.04	-2.40	-5.32
Myocardial infarction	2369.36	2354.16	2346.54	-15.20	-22.83
Congestive heart failure	650.70	610.19	589.74	-40.51	-60.96
Stroke	1304.16	1296.68	1291.43	-7.48	-12.73
**Microvascular**					
Blindness	601.82	595.48	592.69	-6.34	-9.12
End-stage renal disease	184.64	193.94	198.78	9.30	14.14
Amputation	793.61	782.73	777.79	-10.88	-15.81
Ulcer	304.65	287.90	280.04	-16.75	-24.62
Symptomatic hypoglycemia	152807.11	148036.52	143813.75	-4770.59	-8993.36
Severe hypoglycemia	4925.08	4771.44	4622.42	-153.64	-302.65
**Fatal**					
Event related	2958.66	2943.42	2934.86	-15.24	-23.81
Diabetes related	2008.28	1988.35	1977.88	-19.93	-30.40
Other	3923.62	3953.72	3969.79	30.10	46.17
**Total Costs**	**Arm 1: Sitagliptin 100 mg +metformin**	**Arm 2: Semaglutide 0.5 mg +metformin**	**Arm 3: Semaglutide 1 mg +metformin**	**Difference: Arm 2 vs. Arm 1**	**Difference: Arm 3 vs. Arm 1**
**Macrovascular**					
Ischemic heart disease	56,365,873	56,278,312	56,130,648	-87,561	-235,225
Myocardial infarction	100,884,321	99,898,236	99,532,570	-986,085	-1,351,751
Congestive heart failure	17,560,742	16,422,500	15,841,470	-1,138,242	-1,719,272
Stroke	39,208,385	38,843,522	38,579,151	-364,864	-629,235
**Microvascular**					
Blindness	21,556,096	21,227,298	21,117,704	-328,799	-438,392
End-stage renal disease	5,030,814	5,281,358	5,411,921	250,545	381,108
Amputation	12,230,814	11,957,783	11,853,961	-273,031	-376,853
Ulcer	13,401,031	12,597,390	12,259,032	-803,642	-1,141,999
Hypoglycemia	11,416,390	10,766,050	10,173,373	-650,340	-1,243,017
Adverse events	0	0	0	0	0
Pharmacy costs	552,494,106	584,850,432	680,382,745	32,356,327	127,888,639
BMI costs	1,979,909,893	1,603,378,853	1,377,510,607	-376,531,040	-602,399,286
Other costs	23,880,933	23,956,668	23,995,468	75,735	114,535
Total	2,833,939,398	2,485,458,401	2,352,788,650	-348,480,997	-481,150,749

**Cost-Effectiveness (Per Patient)**	**Arm 1: Sitagliptin 100 mg +metformin**	**Arm 2: Semaglutide 0.5 mg +metformin**	**Arm 3: Semaglutide 1 mg +metformin**	**Difference: Arm 2 vs. Arm 1**	**Difference: Arm 3 vs. Arm 1**
Discounted Cost	283,394	248,546	235,279	-34,848	-48,115
Discounted QALYs	12.152	12.234	12.274	0.082	0.122
Discounted Life Years	14.077	14.089	14.095	0.012	0.018
Cost per QALY				-424,428	-394,709
Cost per Life Year				-2,999,029	-2,659,385

BMI body mass index, QALY quality-adjusted life year.

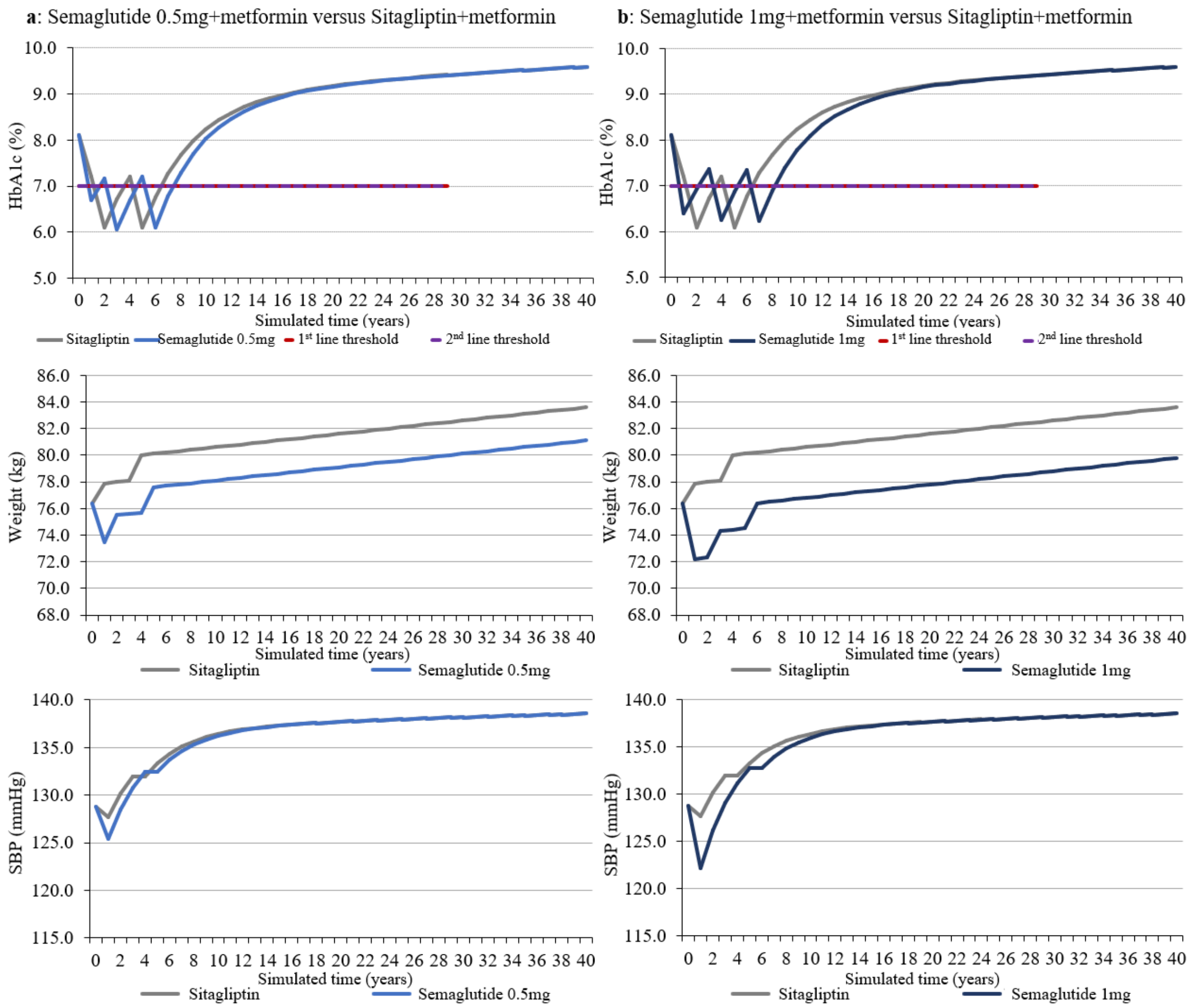

Fig. S1 The trajectories of clinical risk factors in once-weekly semaglutide 0.5 mg + metformin versus sitagliptin + metformin (a) and once-weekly semaglutide 1 mg + metformin versus sitagliptin + metformin (b) over time.

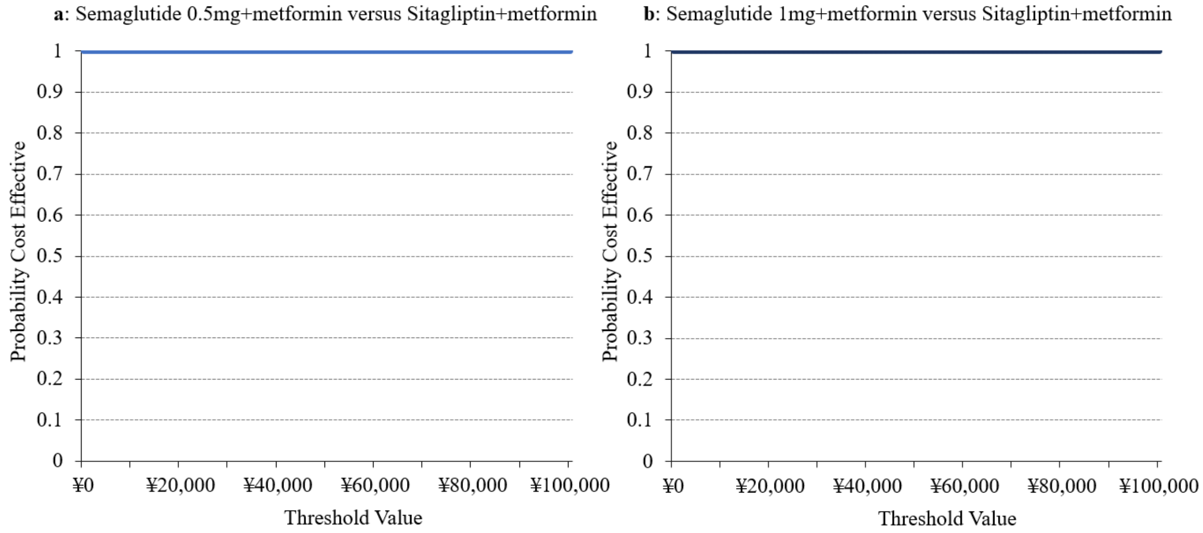

Fig. S2 Cost effectiveness acceptability curves for once-weekly semaglutide 0.5 mg + metformin versus sitagliptin + metformin (a) and once-weekly semaglutide 1 mg + metformin versus sitagliptin + metformin (b).
